# Fusion to the Lysosome Targeting Signal of the Invariant Chain Alters the Processing and Enhances the Immunogenicity of HIV-1 Reverse Transcriptase

**Published:** 2014

**Authors:** E. S. Starodubova, M. G. Isaguliants, Y. V. Kuzmenko, A. A. Latanova, O. A. Krotova, V. L. Karpov

**Affiliations:** Engelhardt Institute of Molecular Biology Russian Academy of Sciences, Vaviolva str., 32, 119991, Moscow, Russia; D.I. Ivanovsky Institute of Virology, Ministry of Health of the Russian Federation, 123098, Moscow, Russia; Department of Microbiology, Tumor and Cell Biology, Karolinska Institutet, 17177, Stockholm, Sweden

**Keywords:** reverse transcriptase, invariant chain, antigen processing, DNA immunization, T-helper immune response

## Abstract

Intracellular processing of the antigen encoded by a DNA vaccine is one of the key steps in generating an immune response. Immunization with DNA constructs targeted to the endosomal-lysosomal compartments and to the MHC class II pathway can elicit a strong immune response. Herein, the weakly immunogenic reverse transcriptase of HIV-1 was fused to the minimal lysosomal targeting motif of the human MHC class II invariant chain. The motif fused to the N-terminus shifted the enzyme intracellular localization and accelerated its degradation. Degradation of the chimeric protein occurred predominantly in the lysosomal compartment. BALB/c mice immunized with the plasmid encoding the chimeric protein demonstrated an enhanced immune response, in the form of an increased antigen-specific production of Th1 cytokines, INF-γ and IL-2, by mouse splenocytes. Moreover, the majority of the splenocytes secreted both cytokines; i.e., were polyfunctional. These findings suggest that retargeting of the antigen to the lysosomes enhances the immune response to DNA vaccine candidates with low intrinsic immunogenicity.

## INTRODUCTION

DNA vaccine-encoded antigens are synthesized in the cellular cytoplasm, processed mainly by proteasomes, and presented on the cell membrane through MHC class I molecules that are recognized by cytotoxic CD8+ T-lymphocytes [1]. At the same time, plasmid DNA-injected mice require MHC class II-dependent activation of CD4+ T-cells to mount a strong immune response [2, 3]. There are now techniques for enhancing the efficacy of DNA vaccines aimed at increasing CD4+ T-cell involvement in the antigen-driven immune response [4, 5]. One of such techniques is based on an enhanced presentation of the plasmid-encoded antigens in the context of MHC class II molecules. The antigenic peptides to be presented on the MHC class II molecules are generated through the lysosomal proteolysis [6]. Antigen presentation by MHC class II molecules can be artificially enhanced by incorporation into the antigen of the lysosomal targeting motives [7].

MHC class II molecules are transported to the lysosomes in association with the invariant chain (Ii) bearing the lysosome-targeting sequence at the N-terminus [6]. The signal peptide is located at the N-terminus (amino acid residues 1–30), with Leu7, Ile8, Pro15, Met16, and Leu17 being the most important residues in the functional context [8-10]. It has been shown that fusion of proteins with this signal sequence promotes their transport to the lysosomes [11-15]. In this work, we constructed a plasmid encoding HIV-1 reverse transcriptase (RT ) of HIV-1 fused to the human MHC class II invariant chain in order to alter RT processing and enhance its immunogenicity, which was reported to be poor [16, 17]. The incorporation of the invariant chain motif shifted the localization of RT towards the lysosomal compartment and increased the rate of its degradation by lysosomal proteases, which led to the augmentation of the immunogenicity of the RT gene in mice.

## EXPERIMENTAL

**Cloning of DNA constructs**

Plasmid pKCMV2RT encoding the HIV-1 HXB2 Reverse Transcriptase (RT ) was generated by recloning the *RT *gene from pCMVRT into the pKCMV plasmid vector using *SalI *and *EcoRI *restriction sites [18] [19]. RT -encoding fragment contained in pCMVRT was amplified by PCR with Pfu-polymerase (Fermentas, Lithuania) using the following primers: RT -SalI-BsiWIF (5'-tcaggtcgactgaacgtacgatgcccattagccctattg-3') and RT -BamHI-EcoRI-R (5'-agtagaattcatgtggatccctagagcactttcctgattccagc- 3').

Plasmid pKCMV2RT -Ii encoding RT fused N-terminally to the human MHC class II invariant chain was designed step-wise from pKCMV2RT . For this, the nucleotide sequence of the human MHC class II invariant chain motif (NM_001025159.1) was generated using synthetic oligonucleotides (Sintol, Russia). The first step involved annealing of the oligonucleotides IC_1–45 (corresponding to the positions 1 to 45 of the invariant chain gene (5'-atggatgaccagcgcgaccttatctccaacaatgagcaactgccc- 3')), IC_46–90 (phosphorylated at the 5’-end and corresponding to the positions 46–90 (5'-atgctgggcc ggcgccctggggccccggagagcaagtgcagccgc-3')) and IC-mid (corresponding to the positions 37 to 71 of the reverse sequence (5'-ggcgccggcccagcatgggcagttgctcattgttg-3')), followed by ligation of a single-stranded break between IC_1–45 and IC_46–90. In the second step, the doublestranded fragment served as a template for PCR . PCR was carried out with Pfu polymerase and IC-F (5'-atccgtcgacatggatgaccagcgcgacc- 3') and IC-R (5'-tgcgcgtacggcggctgcacttgctctc- 3') primers bearing the SalI and BsiWI restriction sites, respectively. Finally, the amplified PCR fragment was cloned into pKCMV2RT at the 5'-end of the *RT *gene, using the SalI and BsiWI sites. This produced an in-frame fusion between Ii and RT . The nucleotide sequence of the cloned fragment was verified by DNA sequencing.

The plasmids used to immunize animals were purified using Plasmid EndoFree kits (QIAGEN , Germany) following the manufacturer’s instructions.

**Preparation and transfection of cell cultures**

Cervical adenocarcinoma cells *(*HeLa*) *were cultured in a DMEM medium (PanEko, Russia) containing 10% fetal bovine serum and a mixture of 100 U/ml penicillin and 100 µl/ml streptomycin. Cells were transfected with plasmids using Lipofectamine LTX (Invitrogen, USA) according to the manufacturer’s manual.

**Quantification of the fusion protein in the transfected cells**

The level of expression of RT variants in the transfected cells were evaluated by immunoblotting. Two days posttransfection, the cells were lysed in a RIPA buffer (10 mm Tris-HCl pH 7.5, 150 mM NaCl, 1% sodium deoxycholate, 1% Triton X-100, 0.1% SDS, 1 mM EDTA). Lysates normalized to the protein content were run on a 10% polyacrylamide gel (PAGE) under denaturing conditions and transferred onto the nitrocellulose membrane (BioRad, USA). To block nonspecific binding sites, the membranes were incubated overnight at 4°C in a PBS-T buffer (80 mM Na_2_HPO_4_, 20 mM NaH_2_PO_4_, 100 mM NaCl, 0.1% Tween-20) supplemented with 5% non-fat milk, followed by incubation with rabbit anti- RT polyclonal antibodies diluted a 1/5000 [20] followed by incubation with the horseradish peroxidase-conjugated goat anti-rabbit IgG antibodies (Jackson, USA) diluted 1/5000. Further, the blots were stripped and re-probed with mouse anti-beta-actin monoclonal antibodies (Sigma, USA) diluted 1/5000 followed by the horseradish peroxidase-conjugated goat anti-mouse antibodies (Jackson, USA) diluted 1/5000. Specific bands on the blots were visualized using a chemiluminescence detection kit (ECL^TM^ plus; Amersham Pharmacia Biotech., USA); the emission from the blots was registered on X-ray films (Fuji Film, Japan). The films were scanned, and the signals were quantified with the ImageJ software (http://rsb.info.nih.gov/ij).

**Half-life of the fusion protein in transfected cells**

The half-life of the fusion protein was evaluated by cycloheximide chase assay as described [21]; the technique had been previously used to estimate the halflife of RT [22]. Two days posttransfection, the cells were mixed with cycloheximide (Sigma, USA) at 100 µg/ml and incubated for 0, 2 and 4 h. After incubation, cells were lysed, lysates were normalized with respect to the protein content and resolved by electrophoresis in 10% SDS-PAAG followed by Western blotting (see section above). The RT content was quantified as described above.

The half-life of RT was determined using the standard half-life equation *N *= *N*_0_ × 2*^t/T^*, where *N*_0_ is the initial protein quantity, *N *is the amount of protein at time* t*, and *T *is the half-life of the protein.

**Inhibition of proteasomal and lysosomal proteolysis in cell culture**

The proteasomal inhibitors MG132 and epoxomicin (Calbiochem, USA) were used at concentrations of 5 and 0.1 µM, respectively. The activity of the lysosomal proteases was inhibited by chloroquine (Sigma, USA) at a concentration of 100 µM. Inhibitors were added to the cell culture 24 h posttransfection. The cells were cocultured with inhibitors for an additional 18 h and then lysed and tested for the presence of the target proteins by immunoblotting.

**Immunostaining of cells**

HeLa cells were grown and transfected on 20 × 20 mm glass cover slips in 6-well plates. At day 2 posttransfection, the cells were fixed with a mixture of acetonemethanol (1 : 1) for 1 h at –20°C. Fixed cells were incubated in a PBS buffer for 30 min at room temperature, followed by sequential incubations with rabbit anti-RT polyclonal antibodies [20] diluted 1/100, swine anti-rabbit IG antibodies conjugated to TR ITC (Dako, Denmark) diluted 1/50, and anti-human lysosomal-associated membrane protein 1 monoclonal antibodies (LAMP1, also known as CD107a) conjugated to FITC (BD Pharmingen, USA) diluted 1/50. All antibodies were diluted in PBS containing 0.5% Tween-20 and 2% bovine serum albumin (BSA). After each incubation, cells were washed three times with PBS. Finally, nuclei were stained for 1 min with 150 mM DAPI (4,6-Diamidino-2-phenylindole, dihydrochloride; Invitrogen, USA). The cover slips were mounted on the glass slides in Vectashield (Vector Laboratories, USA) and viewed under a Leica TC S5 laser confocal microscope (Leica, Germany).

**DNA immunization of mice**

Immunization was performed in BALB/c female mice (8-weeks; Charles River Laboratories, Sandhofer, Germany). Groups of 3-4 mice received plasmids encoding RT -Ii, or RT , or the empty vector. Each mouse received two injections of 10 µg plasmid DNA in 20 µl PBS. Plasmids were injected intradermally with an insulin syringe at the left and right sides of the back near the base of the tail, followed by electroporation of the injected area using DermaVax (Celectis, France) as described [23]. Six days following the first injection, 50 µg of the same plasmid in PBS was administered intramuscularly into the tibialis anterior of the hind limb. At day 28, mice were bled from the tail vein, euthanized and spleens were collected. The immunization experiment was repeated twice.

**FluoroSpot**

The spleens of immunized mice were homogenized individually, and splenocytes were recovered [24]. SplenSplenocytes were incubated in RPMI containing protein RT (12.5 µg/ml) [25, 26], or peptides representing RT amino acids (aa) 375–389 (ITTE SIVIWGKTPKF) or aa 528– 543 (KEKVYLAWVPAHKGIG) at 10 mg/ml. Stimulation with concanavalin A (ConA; 5 µg/ml) served as a positive, and with medium alone, as a negative control. After a 20-h incubation with specific and control antigens, splenocytes were assessed for the level of production of IFN-γ and IL-2. Cytokine secretion was analyzed with the FluoroSpot technique using FluoroSpot plates and a Dual IFN-γ/IL-2 FluoroSpot kit (Mabtech, Sweden) following the manufacturer’s instructions. Cytokine- secreting cells were counted on an AID ELISpot reader (Autoimmun Diagnostika GmbH, Germany).

## RESULTS

**Accumulation and cellular localization of the chimeric reverse transcriptase with an in-built fragment of the MHC class II *invariant chain***

**Fig. 1:**
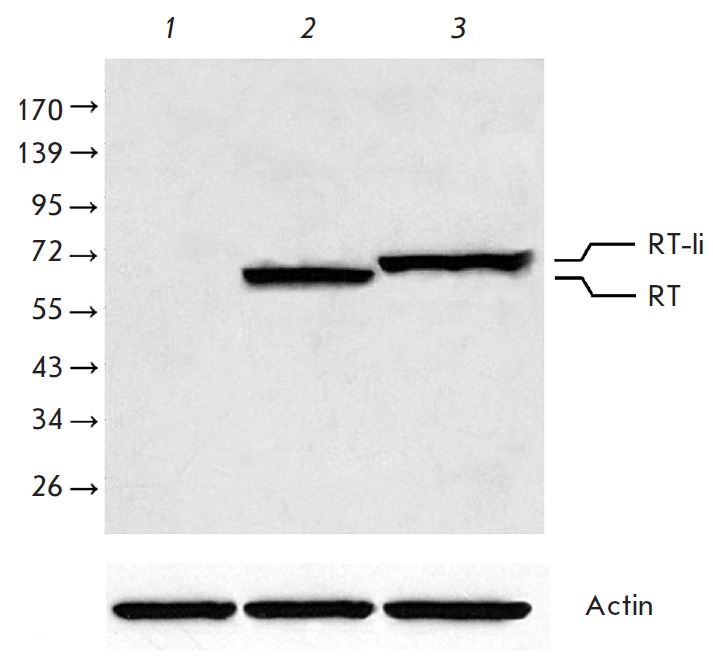
Accumulation of RT and RT-Ii in HeLa cells. Immunoblotting of the lysates of: non-transfected cells (*1*); cells transfected with pKCMV2RT (*2*) or pKCMV2RT-Ii (*3*). Blots were stained with anti-RT polyclonal antibodies. To normalize the signal to the total protein content of the loaded samples, the membranes were stripped and reprobed with anti-actin antibodies. Position of the protein molecular mass standards (in kDa) is indicated to the left

To change the processing of HIV-1 reverse transcriptase, we constructed a plasmid encoding a fusion protein composed of the RT sequence N-terminally fused to the lysosome-targeting signal of the invariant chain (Ii) of human MHC class II, which promotes its transport to the lysosomes through the endoplasmic reticulum (ER ). To accomplish this, we designed a pKCMV2RT -Ii plasmid carrying the *RT *gene with the 5’-end insert of the nucleotide sequence encoding the 30-amino acid signal sequence of Ii. HeLa cells transfected with this plasmid expressed a 68–70kDa protein consistent with the expected molecular mass of RT -Ii chimera (*Fig. 1*, *lane 3*).

**Fig. 2:**
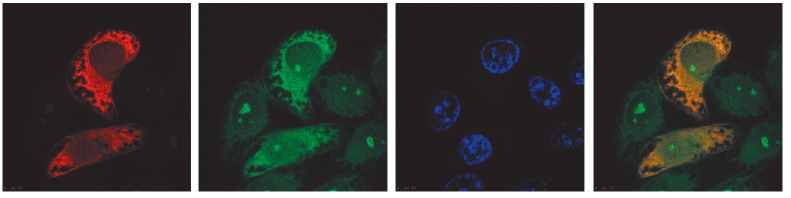
Intracellular localization of the chimeric RT-Ii. HeLa cells were transfected with the pKCMV2RT-Ii plasmid, fixed and stained with anti-RT polyclonal antibodies and secondary TRITC-conjugated antibodies (*1*), anti-human CD107a monoclonal antibodies conjugated to FITC (*2*), or DAPI (*3*). Superposition of images demonstrating RT-specific and CD107a-specific stainings (*4*)

We had previously shown that in cells transfected with the plasmid encoding reverse transcriptase the encoded protein is uniformly distributed throughout the cytoplasm [27]. In this study, immunostaining of HeLa cells transfected with the plasmid encoding the RT -Ii chimera demonstrated a shift from the diffuse to a vesicular pattern (*Fig. 2*). Such a vesicular pattern is indicative of the invariant chain on the way to the endosomal- lysosomal compartment [28]. Co-staining of the transfected cells with anti-RT antibodies and antibodies against the lysosomal-associated membrane protein 1 (LAMP1, CD107a) restricted to the endosomal-lysosomal compartment revealed almost full overlap of the signals (*Fig. 2, field 4*). These findings suggest the invariant chain-driven transport of RT to the lysosomes.

**Incorporation of the invariant chain sequence alters the degradation of RT**

**Fig. 3:**
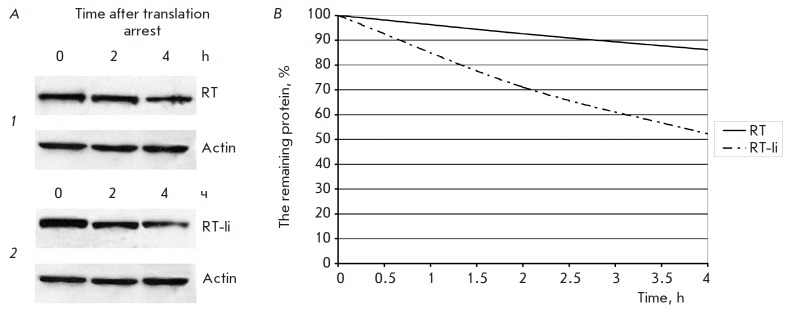
Comparison of RT and RT-Ii degradation in the expressing cells. *A *– Immunoblotting of HeLa cells transfected with pKCMV2RT (*1*) and pKCMV2RT-Ii (*2*) sampled at the given time-points after the addition of cycloheximide (100 μg/ml). Blots were stained with anti-RT polyclonal antibodies. To normalize the signal to the total protein content of the loaded samples, the membranes were stripped and re-probed with anti-actin antibodies. *B *– The kinetics of degradation of RT and RT-Ii

Targeting of reverse transcriptase into lysosomes changes its degradation rate as well as the range of proteases involved in RT degradation. We have previously shown that RT has a half-life of 18 h [29, 30]. The halflife of the RT -Ii chimera assessed after the translation arrest reduced to 4.5 h (*Fig. 3*). Thus, the fusion of RT to the Ii signal sequence caused a four-fold decrease in the protein half-life.

**Fig. 4:**
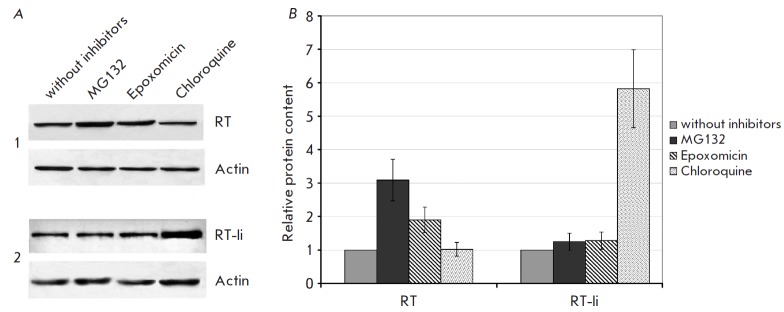
Accumulation of RT and RT-Ii following treatment of expressing cells with proteasomal and lysosomal inhibitors.* A *– immunoblotting of HeLa cells transfected with pKCMV2RT (*1*) and pKCMV2RT-Ii (*2*), after co-culturing for 18 h with MG132 (5 μM), epoximicine (0.1 μM), chloroquine (100 μM), or in the medium alone. Blots were stained with anti-RT antibodies. To normalize the signal to the total protein content of the loaded samples, the membranes were stripped and re-probed with anti-actin antibodies. *B *– The diagram showing the relative content of RT in cells treated with the proteasomal or lysosomal inhibitors as compared to the untreated cells

The role of proteasomal and lysosomal pathways in the processing of the RT -Ii chimera was examined using specific inhibitors. The in-put of proteasome into RT -Ii degradation was assessed with the help of MG132 and epoxomicin; and of lysosome, with the help of chloroquine. The transfected Hela cells were co-cultured with the inhibitors for 18 h and then subjected to immunoblotting to evaluate the accumulation of the protein in comparison with that in the untreated cells (*Fig. 4*). Lysosomal inhibitor chloroquine had no effect on the accumulation of RT but increased the amount of RT -Ii in the cells by more than six-fold (*Fig. 4*). At the same time, RT -Ii, in contrast to RT , appeared to be insensitive to the proteasomal inhibitors (*Fig. 4*).

**Introduction of the lysosomal targeting signal enhances the immunogenicity of RT**

**Fig. 5:**
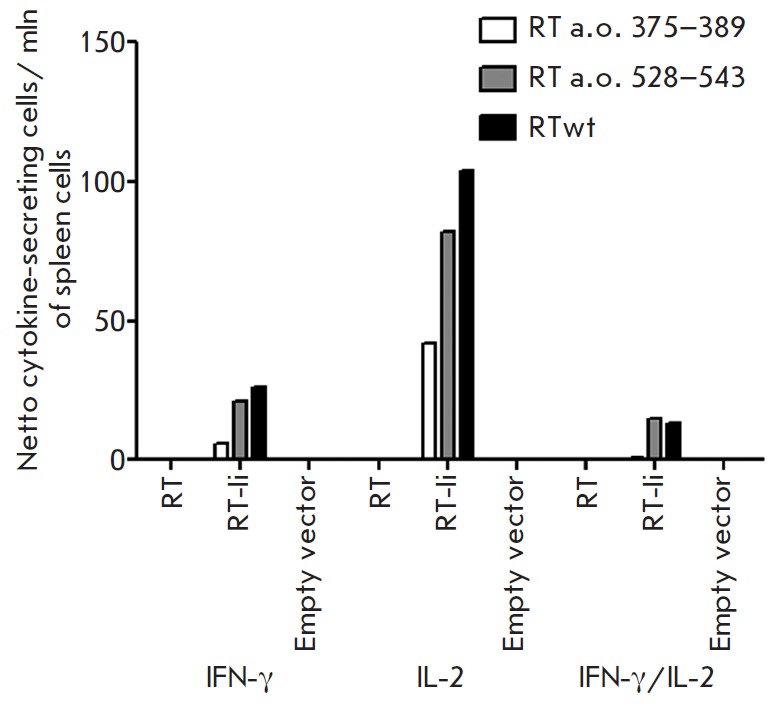
Immune response of mice immunized with the plasmids encoding RT and RT-Ii. Mice were immunized with the plasmids pKCMV2RT and pKCMV2RT-Ii, encoding RT and RT-Ii, respectively, or an empty vector. After completion of immunization, mice were sacrificed, and their splenocytes were harvested and stimulated with reverse transcriptase (RTwt) or RT-derived peptides (RT 375–389 or RT 528–543). The number of splenocytes producing IFN-γ and IL-2 in response to specific antigen stimulation was assessed by FluoroSpot assay as the average number of signal-forming units (sfu) per mln cells with the background secretion in the media subtracted

BALB/c mice were primed by the intradermal injection of the RT -Ii encoding plasmid, followed by electroporation. Five days after the first injection, they received a boost with the same plasmid by the intramuscular route. The control mice received either a plasmid encoding the parental *RT *gene, or an empty vector. The immune response was assessed by the capacity of murine splenocytes to produce IFN-γ and IL-2 after* in vitro *stimulation with the protein RT or RT -derived peptide representing its immunodominant epitopes at amino acid residues 375–389 and 528–543 [31, 32]. IFN-γ, IL-2, or IFN-γ / IL-2 producing cells were identified with the Fluorospot assay (*Fig. 5*). A specific production of IFN-γ and IL-2 in response to stimulation with the protein or peptide was observed only in mice immunized with the plasmid encoding the RT -Ii chimera. Most of the cells producing IFN-γ also produced IL-2, which demonstrated their polyfunctionality. No specific cytokine production was observed in mice receiving the RT gene or the empty vector (*Fig. 5*).

## DISCUSSION

CD4+ T-cells play an important role in mounting a potent immune response, stimulating both the cellular*-*( Th1-type CD4+ T-cells) and humoral-(Th2-type) mediated immunity. CD4+ T-cells are activated after recognition by their receptors of the complexes of antigen- derived peptides with the MHC class II molecules on the surface of antigen-presenting cells [33]. Peptides bound by MHC class II molecules mainly originate from the exogenous proteins that are taken up by endocytosis and transported to lysosomes [34]. However, there are mechanisms that load MHC class II molecules with peptides derived from the self-proteins. These peptides are generated by the processing of the proteins associated with the lysosomes [35, 36]. Based on these findings, we attempted to fuse our antigen with a natural signal sequence targeting proteins to the lysosome and thus re-direct it into the lysosomal compartment for processing and presentation in complex with MHC class II molecules [7].

Attempts to re-route antigens to enhanced presentation by MHC class II molecules have been made, which employed the sorting signal of LAMP-1 [37] and the AP3*-*binding motif of LIMP II [38]. Studies of the immunogenicity of such chimeras delivered into animals as DNA vaccines showed their capacity to elicit stronger immune responses than the parental immunogens [24, 39, 40]. Immunization by genes encoding lysosomal- targeted fusion proteins led to both an enhanced production by B cells of the protective antibodies, and an enhanced cytolytic activity. Also, in most cases, the longevity of the immune response induced by the lysosome- targeted antigen exceeded the longevity of the response to the parental antigen [7].

Fusion of the invariant chain to recombinant antigens demonstrated the potential to increase both the immunogenicity and the duration of a protective immune response to the prototype DNA vaccines in laboratory animals. DNA constructs encoding a chimera composed of Ii and the lymphocytic choriomeningitis virus glycoprotein showed an enhanced capacity to activate both CD4+ and CD8+ T-cells [12]. Single administration of this plasmid to mice conferred protection against a lethal challenge with this virus [12]. The efficacy of such treatment was also reported in DNA immunization of large animals, which permitted construction of a DNA vaccine encoding a chimeric protein composed of the *Anaplasma marginale *major surface protein and the bovine invariant chain fragment [13]. A single injection of this DNA construct into calves elicited production of IgG antibodies against *Anaplasma* and increased the proliferation of CD4+ T-cells, accompanied with antigen specific-secretion of IFN-γ. This DNA immunization proved to be sufficient to mount immune memory for a rapid recall response upon antigen re-exposure [13].

In this work, we designed a DNA construct encoding the HIV-1 subtype B reverse transcriptase N-terminally fused to the lysosomal targeting signal of the human MHC class II invariant chain. The chimeric protein was shown to accumulate in the vesicular compartments such as ER , Golgi apparatus, and endosomal/lysosomal compartment. The introduction of the Ii signal resulted in a significant (four-fold) decrease of the half-life of the chimeric protein as compared to the parental RT . Proteasome inhibitors had no effect on the cellular accumulation of the chimera. At the same time, treatment of cells expressing RT -Ii with the lysosomal inhibitor led to a significant accumulation of the chimeric protein. Overall, the attachment to RT of the lysosomal targeting signal of human MHC class II invariant chain induced a shift from the proteasomal to the lysosomal route of degradation.

Mice immunized with the plasmid encoding the chimera mounted antigen-specific IFN-γ and IL-2 responses, whereas the parental RT was nonimmunogenic. Thus, insertion of the fragment encoding the lysosomal targeting sequence of the invariant chain allowed us to overcome the poor immunogenicity of the* RT *gene immunogen. ;

Of note, most of the splenocytes of the RT -Ii immunized mice were able to secret both IFN-γ and IL-2. IFN-γ secretion is an important parameter that demonstrates an onset of the protetive immune response against viral infection. IL-2 plays an essential role in the expansion of the memory T-cells critical for longterm protective immunity [41]. Most of the epitopespecific cytotoxic lymphocytes produce IFN-γ; a proportion of these cells secretes also IL-2 and/or TN F-α, i.e. are polyfunctional [42]. These cells are required for an efficient control of the infections, as well as for the generation of a protective response following vaccination [43, 44]. The approach to DNA-vaccine design utilized herein ensures the generation of a polyfunctional immune response, allowing to build such a response against vaccine candidates with intrinsically poor immunogenicity.

## CONCLUSIONS

Fusion to a sequence of the human invariant chain carrying the lysosomal targeting signal was used to improve the immunogenic performance of a prototype DNA-vaccine based on HIV-1 reverse transcriptase. The lysosome-targeting sequence inserted at the Nterminus of HIV-1 RT changed both its cellular localization and the degradation pathway. This modification allowed to overcome the poor immunogenicity of reverse transcriptase as DNA-immunogen, generating a potent antigen-specific immune response in mice. The improved HIV-1 RT -based DNA construct could be included into multi-gene DNA vaccines against HIV-1 to enhance their efficacy.

## Abbreviations

HIV: Human immunodeficiency virus
MHC: major histocompatibility complex
ER: endoplasmic reticulum
Ii: MHC class II-associated invariant chain
IFN-γ: interferon-gamma
IL-2: Interleukin 2
RT: reverse transcriptase
